# Effect of fasting blood glucose on risk of new‐onset hypertension in rural Chinese population: a 15-year follow-up cohort

**DOI:** 10.1186/s12872-021-02336-4

**Published:** 2021-11-08

**Authors:** Jing Liu, Nan N. Cheng, Zi Y. Zhou, Yue Zhang, Jie Yang, Li S. Liu, Yun Song, Xiao Huang, Gen F. Tang, Bin Y. Wang, Xian H. Qin, Xi P. Xu, Xiang Q. Kong

**Affiliations:** 1 Department of Cardiology, The First Affiliated Hospital of Nanjing Medical University, 300 Guangzhou Road, Nanjing, 210029 Jiangsu People’s Republic of China; 2 State Key Laboratory of Natural Medicines, Research Center of Biostatistics and Computational Pharmacy, China Pharmaceutical University, Nanjing, 210009 People’s Republic of China; 3 Beijing Advanced Innovation Center for Food Nutrition and Human Health, College of Food Science and Nutritional Engineering, China Agricultural University, Beijing, People’s Republic of China; 4 School of Health Administration, Anhui Medical University, Hefei, People’s Republic of China; 5 Department of Cardiovascular Medicine, Second Affiliated Hospital of Nanchang University, Nanchang, People’s Republic of China; 6 National Clinical Research Study Center for Kidney Disease, The State Key Laboratory for Organ Failure Research, Renal Division, Nanfang Hospital, Southern Medical University, Guangzhou, People’s Republic of China

**Keywords:** Fasting blood glucose, Total cholesterol, Hypertension, Risk

## Abstract

**Background:**

The purpose of this study was to examine the correlation between fasting blood glucose and new-onset hypertension and examine any synergistically effect modification with multiple risk factors.

**Methods:**

We conducted post-hoc analyses of repeated-measures data in the original Dongzhi osteoporosis cohort study. In total, 3985 participants without hypertension aged 25–64 years were included in the current analyses. Generalized estimating equation models were used to assess the relationship between fasting blood glucose and risk of new-onset hypertension after adjusting for pertinent covariates and autocorrelations among siblings.

**Results:**

393 men (19.4%) and 398 women (20.3%) without hypertension at the baseline developed hypertension by the end of the study period. Compared to lower baseline fasting blood glucose levels (Q1–Q3: < 5.74 mmol/L; clinical cut points: < 5.6 mmol/L), higher baseline fasting blood glucose levels (Q4: ≥ 5.74 mmol/L; clinical cut points: ≥ 5.6 mmol/L and < 7.0 mmol/L) increased the risk of new-onset hypertension significantly [(OR: 1.54, 95% CI 1.19–1.98, P < 0.001); (OR: 1.38, 95% CI 1.09–1.75, P = 0.008)] in women. Additionally, a stronger significant association was found in women with elevated fasting blood glucose on risk of new-onset of hypertension with higher total cholesterol (≥ 5.2 mmol/L) [(OR: 2.76; 95% CI: (1.54, 4.96), P < 0.001)]. However, no association was found between fasting blood glucose and risk of new-onset hypertension in men.

**Conclusions:**

High fasting blood glucose may be significantly associated with risk of new-onset hypertension in Chinese women, especially in women with higher total cholesterol. Further randomized studies are needed to confirm our findings.

## Background

It is well known that the high prevalence of hypertension in the general population remains the major cause of cardiovascular disease (CVD) and all-cause death in the world. According to 2018 ESC/ESH Guidelines for the management of arterial hypertension [1], the overall prevalence of hypertension is approximately 30–45% of the world’s adult population (24% in men and 20% in women, respectively) in 2015, regardless of in lower, middle, and higher resource community. The current hypertension epidemic is prone to increases the risk of cardiovascular complications and its associated medical burden; therefore, more attention needs to be placed on finding effective methods that help determine high-risk individuals in order to modify these trends.

Previous studies have shown that a cluster of risk factors, such as age, resting heart rate (RHR), overweight or obesity, dyslipidemia, hyperuricemia, impaired glucose regulation, and estimated glomerular filtration rate (eGFR) were additively associated with new-onset hypertension [2]. Recently, the correlation between blood glucose and hypertension has been a research focus. In 2003, the American Diabetes Association (ADA) recommended impaired fasting glucose (IFG), a type of prediabetes defined as the patient’s fasting blood glucose range of 5.6–6.9 mmol/L, that be classified as impaired glucose regulation (IGR) [3]. A Korean community cohort study demonstrated that IFG did not predict risk of hypertension [4]. However, Kuwabara et al. [5] recently suggested that elevated fasting blood glucose might be an independent risk factor for developing hypertension in Japan. The extent to which the relationship between fasting blood glucose and the risk of new-onset hypertension is affected by above-listed confounding factors, therefore, this issue remains controversial. Determining fasting blood glucose levels that are associated with the incidence of hypertension would help minimize the risk and inform clinicians on medical prevention strategies to reduce adverse health issues related to fasting blood glucose.

In this study, we aimed to investigate whether elevated fasting blood glucose acts as an independent risk factor for developing hypertension in a non-hypertensive Chinese rural population and examine any possible effect modifiers using data from a post-hoc analysis of a large-scale community‐based cohort study.

## Methods

### Study design and participants

This study utilized part of data from a post-hoc analysis of a large community‐based osteoporosis cohort study in a rural area (Dongzhi), Anhui province, in Eastern China [6]. Data were obtained at baseline in 2003 and follow up visits were conducted in 2014, 2017 and at the study’s conclusion in 2018. A total of 18,265 participants actually registered, were enrolled to conducted questionnaire surveys, physical examinations, and biological sample collection at baseline in 2003 with a mean follow-up interval of 14.1 years (as detailed below).

**Fig. 1:**
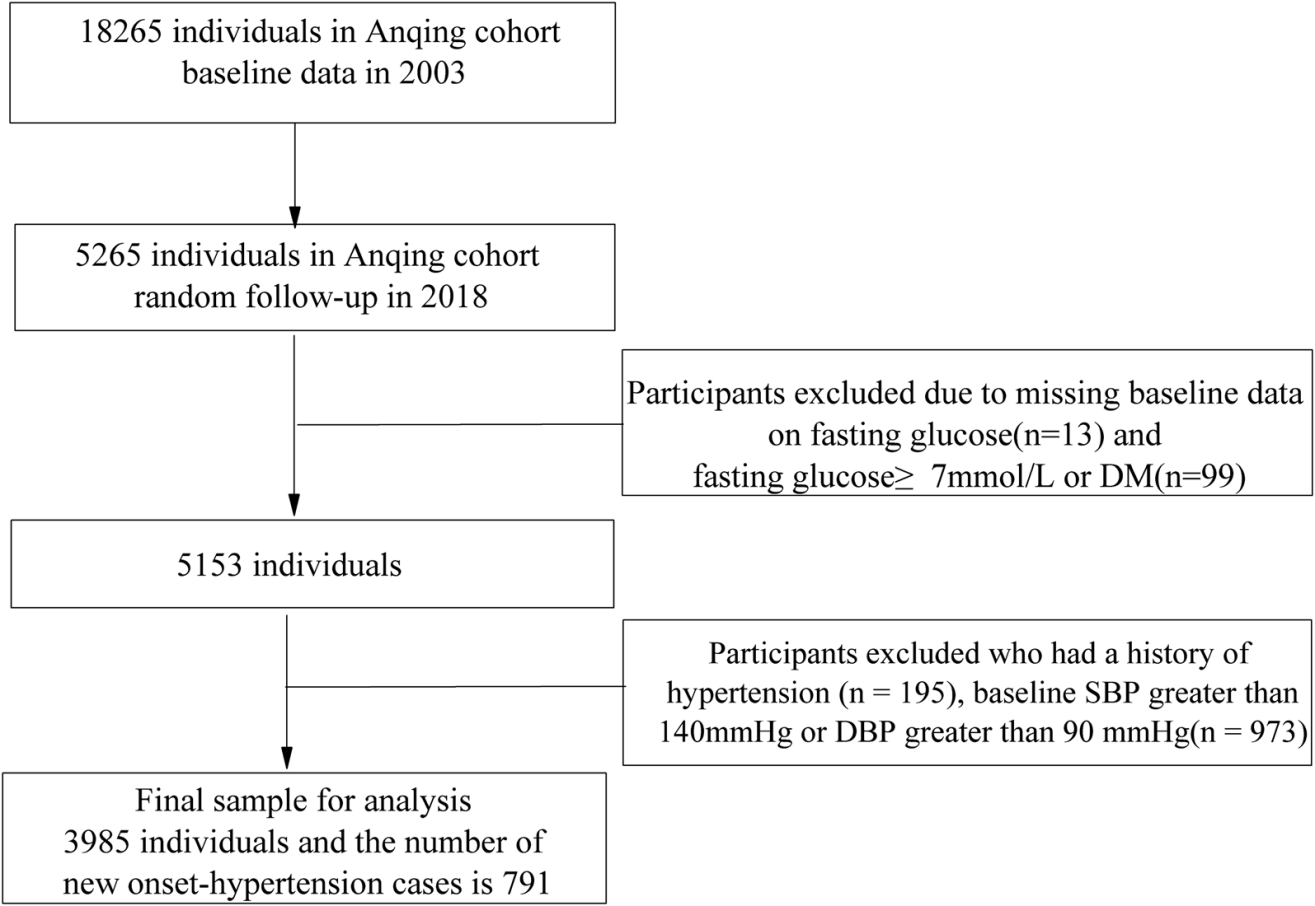
Flow of the participants in the current study

Data on new-onset hypertension and fasting blood glucose were obtained by telephone or face-to-face interviews or measurements with family members during follow-up, so, therefore, we obtained these data from 5265 participants at the end of the follow-up study in 2018, whose Identity (ID) were matched with these at baseline in 2003. Anyone with chronic infections, renal failure, history of hypertension, diabetes mellitus (DM), rickets or other metabolic bone diseases, chronic glucocorticoid use, thyrotoxicosis, and premenopausal women who were uncertain of their pregnancy status at the time of enrollment was not enrolled in this study. 13 participants were excluded from analysis due to missing data on fasting blood glucose. 99 participants with fasting blood glucose concentration ≥ 7.0 mmol/L or history of DM at baseline, 195 participants with a history of hypertension at baseline, and 973 participants who had been diagnosed with hypertension at the start of the study, were all excluded. Therefore, a total of 3,985 participants were included in the final analysis and the number of new-onset hypertension participants was 791. The flow of the participants enrolled in the current study is shown in Fig. 1.

The complete examination includes completion of a survey questionnaire face to face, administered by professionally trained investigators, that included information on demographic characteristics, personal and family chronic diseases histories, medical history, health-related lifestyle factors, dietary intake, and any uncommon medical events that occurred during the study period. In addition, participants underwent a thorough physical examination, including the measurements of height, weight, waist circumference, hip circumference, and blood pressure (BP) at baseline and at the study’s conclusion in 2018. Body mass index (BMI) was calculated as weight in kg divided by the square of height in m^2^. Waist-to-height ratio (WHtR) was calculated as the waist circumference divided by the height circumference. Waist-to-hip ratio (WHR) was calculated as the waist circumference divided by the hip circumference. A 10 ml blood sample was also collected from all participants.

The authors state that all supporting data will be made available. The study was conducted with the approval of the independent ethics review board of Anhui Medical University (The committee’s reference number is 1005 2003-8-11). All procedures were performed in accordance with ethical standards. The research was performed in accordance with the Helsinki Declaration of 1964 and its later amendments and applicable the relevant guidelines and regulations. Written informed consent was obtained from each participant.

### Blood pressure measurement

After a 5–10 min rest period, blood pressure measurements were obtained from seated participants using the conventional cuff method, in a quiet environment. The means of three SBP and DBP measurements were used for data analysis. Hypertension was defined as SBP ≧ 140 mmHg and/or DBP ≧ 90 mmHg or use of antihypertensive medication according to National High Blood Pressure Education Program [7].

### Blood biochemical measurements

Fasting blood samples at baseline and at the exit visit were collected from each participant to measure fasting blood glucose and serum lipids (total cholesterol (TC), triglycerides (TG), and high-density lipoprotein cholesterol (HDL-C)). Samples were stored in aliquots at − 80 °C and enzymatic measurements were analyzed using a Cobas Integra Roche analyzer (Roche, Indianapolis, IN).

### Glucose-status classification

Baseline fasting blood glucose concentrations were assessed as a continuous variable, as quartiles, and as a binary variable. For women the quartiles were: Quartiles 1 (< 5.04 mmol/L), Quartiles 2 (5.04 to < 5.36 mmol/L), Quartiles 3 (5.36 to < 5.74 mmol/L), and Quartiles 4 (≥ 5.74 mmol/L), and for men they were: Quartiles 1 (< 4.99 mmol/L), Quartile 2 (4.99 to < 5.33 mmol/L), Quartile 3 (5.33 to < 5.70 mmol/L), and Quartile 4 (≥ 5.70 mmol/L). As a binary variable, the clinical cut point for fasting blood glucose concentration was < 5.6 mmol/L, and ≥ 5.6 mmol/L and < 7.0 mmol/L.

### Lipids-profile classification

Dyslipidemia was defined as TC ≥ 5.2 mmol/L, TG ≥ 1.7 mmol/L, and/or HDL-C levels < 1.04 mmol/L or lipid levels within the normal range through the treatment of lipid lowering drugs according to the recommended criteria for Chinese adults in guideline [8]. Baseline lipid levels including TC, TG, HDL-C were assessed as binary variables by clinical cut-points.

### Statistical analysis

Means ± standard deviation (SD) or medians (25th percentile–75th percentile) and frequency with percentages were presented for comparisons of population demographic characteristics within groups. Age, SBP, DBP, BMI, waist circumference and hip circumference, WHR, and WHtR were analyzed as continuous variables, whereas sex, smoking and drinking status were analyzed as categorical variables. Data on fasting glucose, TC, TG, and HDL-C were presented as medians. Generalized Estimating Equation (GEE) was used to evaluate association between baseline fasting blood glucose level and new-onset hypertension. Variables in the stratified analysis included age, BMI, TC, TG, HDL-C, smoking status and drinking status, and accounting for autocorrelations among multiple family members.

All statistical analyses were performed by EmpowerStats (http://www.empowerstats.com) and R software, version 3.5.1 (http://www.R-project.org/). A P < 0.05 was considered to be statistically significant in all analyses.

## Results

### Baseline clinical characteristics of the study participants

Table 1 shows the baseline characteristics of all participants with or without new-onset hypertension by sex. A total of 3,985 participants (2029 men and 1956 women) were included in this study. Of these, 393 males (19.4%) and 398 females (20.3%) developed hypertension during the follow-up period. Women with new onset hypertension were more likely to be older and had higher values of fasting blood glucose, TC, TG, BMI, waist circumference, and WHtR. In comparison, BMI, waist circumference, WHtR, TC, and TG values were significantly higher in men with new-onset hypertension; however, age and fasting blood glucose levels showed no significant differences compared with those without hypertension.

**Table 1:** Baseline characteristics of the study participants by new onset-hypertension

Variables	Male			Female		
	New onset-hypertension		*P value*	New onset-hypertension		*P value*
	No (n = 1636)	Yes (n = 393)		No (n = 1558)	Yes (n = 398)	
Age, y	47.5 ± 7.2	47.4 ± 6.9	0.807	44.7 ± 6.9	46.0 ± 7.2	0.001
SBP, mmHg	113.9 ± 11.6	119.8 ± 10.2	< 0.001	113.3 ± 11.6	119.3 ± 11.4	< 0.001
DBP, mmHg	73.7 ± 8.2	77.6 ± 6.6	< 0.001	72.6 ± 7.9	76.0 ± 7.3	< 0.001
BMI, kg/m^2^	21.0 ± 2.2	21.7 ± 2.7	< 0.001	21.7 ± 2.6	22.6 ± 2.9	< 0.001
Waist circumference, cm	74.2 ± 7.3	76.7 ± 8.2	< 0.001	72.3 ± 7.6	75.2 ± 8.3	< 0.001
WHR, waist/hip	0.85 ± 0.15	0.86 ± 0.06	0.225	0.8 ± 0.2	0.8 ± 0.1	0.242
WHtR, waist/height	0.46 ± 0.04	0.47 ± 0.05	< 0.001	0.5 ± 0.0	0.5 ± 0.1	< 0.001
**Laboratory results**						
Fasting glucose, mmol/L^†^	5.3 (5.0–5.7)	5.3 (5.0–5.7)	0.883	5.3 (5.0–5.7)	5.4 (5.1–5.8)	< 0.001
Total cholesterol, mmol/L^†^	4.2 (3.8–4.8)	4.4 (3.8–4.9)	0.037	4.3 (3.8–4.9)	4.4 (3.9–5.0)	0.016
Triglycerides, mmol/L^†^	0.9 (0.7–1.2)	1.0 (0.8–1.5)	< 0.001	1.1 (0.9–1.5)	1.2 (0.9–1.6)	< 0.001
HDL, mmol/L^†^	1.4 (1.2–1.6)	1.3 (1.1–1.6)	0.037	1.4 (1.1–1.6)	1.3 (1.1–1.6)	0.229
Smoking status, n (%)			0.049			0.296
Never	342 (21.0)	74 (18.9)		1484 (95.6)	381 (96.2)	
Former	126 (7.7)	45 (11.5)		5 (0.3)	3 (0.8)	
Current	1161 (71.3)	273 (69.6)		64 (4.1)	12 (3.0)	
Drinking status, n (%)			0.001			0.125
Never	982 (60.2)	197 (50.3)		1511 (97.5)	384 (96.7)	
Former	31 (1.9)	12 (3.1)		1 (0.1)	2 (0.5)	
Current	617 (37.9)	183 (46.7)		37 (2.4)	11 (2.8)	

BMI, body mass index; SBP, systolic blood pressure; DBP, diastolic blood pressure; HDL-C, high-density lipoprotein cholesterol; WHR, Waist-to-Hip Ratio; WHtR, Waist-to-Height Ratio

For continuous variables, values are presented as mean ± SD. For categorical variables, values are presented as n (%)

^†^Lab values are presented as median (IQR)

### Relationship between fasting glucose and new-onset hypertension stratified by sex

**Fig. 2:**
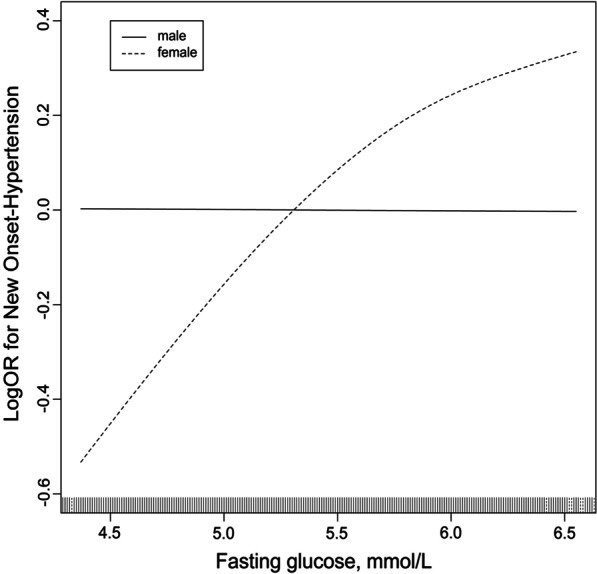
The association between baseline fasting glucose and risk of New-Onset hypertension stratified by sex. Model adjusted for age, body mass index, total cholesterol, triglycerides, high density lipoprotein cholesterol, and smoking and drinking status

We performed univariate logistic regression analyses and calculated to evaluate the association between baseline fasting blood glucose levels and risk of new-onset hypertension (logOR), stratified by sex as shown in Fig. 2. Model was adjusted for major variables which included age, BMI, TC, TG, HDL-C, and smoking and drinking status. The dotted line, that represents females, shows that the risk of new-onset hypertension rises with increasing baseline fasting blood glucose levels after adjustments for confounding covariates. In contrast, the solid line shows that there was no association after adjustments for confounding covariates in males.

This finding was further evaluated by GEE model via odds ratios (OR) and 95% confidence intervals (CIs) as shown in Table 2. In women, when fasting blood glucose was assessed as quartiles, compared with quartile 1, quartiles 2–4 had an increased risk of new-onset hypertension with ORs and 95% CIs of 1.46 (1.03, 2.07), 1.37 (0.96, 1.95), and 1.96 (1.40, 2.75), respectively. A similar result was obtained when Q1, Q2 and Q3 were combined (Q1–Q3: < 5.74 mmol/L), where the risk for those in Q4 significantly increased by 54% (OR: 1.54, 95% CI 1.19–1.98, P < 0.001) compared to Q1–Q3. When fasting blood glucose was modeled as a binary variable according to clinical cut points, compared to the reference group (< 5.6 mmol/L), the risk of new-onset hypertension in women with fasting blood glucose ≥ 5.6 mmol/L and < 7.0 mmol/L, significantly increased by 38% (OR: 1.38, 95% CI 1.09–1.75, P = 0.008). However, there was not statistically significant association between fasting blood glucose and risk of new-onset hypertension in men (Quartiles: P = 0.678, and clinical cut points: P = 0.551). A 2-way interaction test of sex and fasting glucose as quartiles on new-onset hypertension was significant (P = 0.002, Table 2).

**Table 2:** The association between fasting glucose and new onset-hypertension

	N	Case (%)	Crude model	Adjusted model*	*P value*
			OR (95% CI)	OR (95% CI)	
**Fasting glucose, mmol/L**					
**Male**					
Continuous^†^	2029	393 (19.3)	0.99 (0.80, 1.21)	0.96 (0.78, 1.20)	0.739
Quartiles					
Q1 (< 4.99)	503	98 (19.5)	Ref	Ref	
Q2 (4.99–5.33)	509	96 (18.9)	0.94 (0.69, 1.29)	0.95 (0.69, 1.30)	0.738
Q3 (5.33–5.70)	508	100 (19.7)	0.94 (0.68, 1.29)	0.91 (0.66, 1.27)	0.592
Q4 (≥ 5.70)	509	99 (19.5)	0.95 (0.69, 1.30)	0.90 (0.64, 1.26)	0.534
Quartiles					
Q1–Q3 (< 5.70)	1520	294 (19.3)	Ref	Ref	
Q4 (≥ 5.70)	509	99 (19.4)	0.99 (0.76, 1.28)	0.94 (0.72, 1.24)	0.678
Categories					
< 5.6	1406	274 (19.5)	*Ref*	*Ref*	
≥ 5.6	623	119 (19.1)	0.95 (0.74, 1.21)	0.93 (0.72, 1.19)	0.551
**Female**					
Continuous^†^	1956	398 (20.3)	1.51 (1.24, 1.85)	1.47 (1.18, 1.82)	< 0.001
Quartiles					
Q1 (< 5.04)	482	70 (14.5)	*Ref*	*Ref*	
Q2 (5.04–5.36)	482	100 (20.8)	1.55 (1.11, 2.17)	1.46 (1.03, 2.07)	0.032
Q3 (5.36–5.74)	499	100 (20.0)	1.46 (1.04, 2.04)	1.37 (0.96, 1.95)	0.080
Q4 (≥ 5.74)	493	128 (26.0)	2.07 (1.49, 2.86)	1.96 (1.40, 2.75)	< 0.001
Quartiles					
Q1–Q3 (< 5.74)	1463	270 (18.5)	*Ref*	*Ref*	
Q4 (≥ 5.74)	493	128 (26.0)	1.55 (1.22, 1.98)	1.54 (1.19, 1.98)	< 0.001
Categories					
< 5.6	1307	240 (18.4)	*Ref*	*Ref*	
≥ 5.6	649	158 (24.4)	1.42 (1.13, 1.79)	1.38 (1.09, 1.75)	0.008

*Adjusted for age, body mass index, total cholesterol, high density lipoprotein, triglycerides, smoking status and drinking status

^†^A 2-way interaction test of sex and fasting glucose as quartiles on new onset-hypertension was significant (P = 0.002)

### Risk factors-stratified analyses of fasting glucose on new-onset hypertension for men and women

Since the effects of fasting blood glucose on new-onset hypertension risk in women and men were significantly different, further stratified analyses by sex were performed to assess the effects of fasting blood glucose on the risk of new-onset hypertension in various subgroups (Table 3a, b). As shown in Table 3a, the subgroup interaction analyses showed that for every increase of 1 mmol/L fasting blood glucose, the risk of new onset hypertension increased significantly in women (P value for interaction was 0.033). In further analyses, a 176% increased risk of new onset hypertension was seen for those in the high TC group (≥ 5.2 mmol/L) [(OR:2.76 (1.54, 4.96), P value was < 0.001)]; however, a 34% increased risk of new onset hypertension was seen for those in the low TC group (< 5.2 mmol/L) [(OR:1.34 (1.00, 1.78), P value was 0.047)]. TC showed a significant interaction with fasting blood glucose regarding risk of new-onset hypertension for women. Separate subgroup analyses were conducted in men that used the same risk-factors variables as for women, plus smoking status and drinking status, but none of the known risk-factor variables, showed a significant interaction with fasting blood glucose for new-onset hypertension risk.

**Table 3:** Risk factors stratified analyses of fasting glucose on new onset-hypertension in (a) females and (b) males

	Fasting glucose, mmol/L				*P for interaction*
	N	Events (%)	OR (95% CI)	*P*	
*(a)*					
**Age**					0.987
< 50	1478	276 (18.7)	1.50 (1.17, 1.92)	0.002	
≥ 50	478	122 (25.5)	1.48 (0.99, 2.23)	0.058	
**Total cholesterol, mmol/L (Clinical cut point)**					0.033
< 5.2	1672	324 (19.4)	1.34 (1.00, 1.78)	0.047	
≥ 5.2	284	74 (26.1)	2.76 (1.54, 4.96)	< 0.001	
**High density lipoprotein cholesterol, mmol/L**					
**(Clinical cut point)**			0.132		
< 1.04	288	61 (21.1)	2.17 (1.24, 3.82)	0.007	
≥ 1.04	1668	337 (20.2)	1.34 (1.07, 1.70)	0.013	
**Triglycerides, mmol/L (Clinical cut point)**			0.522		
< 1.7	1611	305 (18.9)	1.51 (1.18, 1.92)	0.001	
≥ 1.7	345	93 (27.0)	1.24 (0.78, 1.96)	0.361	
**Body mass index, kg/m**^**2**^				0.281	
Low (< 21.64)	975	152 (15.6)	1.66 (1.22, 2.26)	0.001	
High (≥ 21.64)	973	246 (25.3)	1.35 (1.00, 1.82)	0.048	
**Waist, circumference**					0.179
Low (< 72)	989	152 (15.4)	1.67 (1.21, 2.31)	0.002	
High (≥ 72)	967	246 (25.4)	1.32 (0.99, 1.76)	0.058	
*(b)*					
**Age**					0.488
< 50	1210	235 (19.4)	0.93 (0.71, 1.23)	0.622	
≥ 50	819	158 (19.3)	1.06 (0.76, 1.48)	0.733	
**Total cholesterol, mmol/L (Clinical cut point)**					0.552
< 5.2	1765	334 (18.9)	0.99 (0.78, 1.24)	0.909	
≥ 5.2	264	59 (22.3)	0.84 (0.48, 1.49)	0.556	
**High density lipoprotein cholesterol, mmol/L**					
**(Clinical cut point)**			0.940		
< 1.04	255	53 (20.8)	0.97 (0.56, 1.67)	0.903	
≥ 1.04	1774	340 (19.2)	0.96 (0.76, 1.21)	0.734	
**Triglycerides, mmol/L (Clinical cut point)**			0.187		
< 1.7	1774	317 (17.9)	1.02 (0.80, 1.30)	0.854	
≥ 1.7	255	76 (29.8)	0.75 (0.46, 1.20)	0.229	
**Body mass index, kg/m**^**2**^				0.055	
Low (< 20.85)	1013	162 (16.0)	1.26 (0.90, 1.76)	0.171	
High (≥ 20.85)	1012	230 (22.7)	0.82 (0.62, 1.08)	0.158	
**Waist, circumference**					0.629
Low (< 73)	1037	167 (16.1)	1.01 (0.71, 1.44)	0.964	
High (≥ 73)	992	226 (22.8)	0.93 (0.72, 1.21)	0.610	
**Smoking status**					0.404
Never	416	74 (17.8)	0.85 (0.55, 1.31)	0.453	
Ever	1605	318 (19.8)	0.99 (0.77, 1.27)	0.938	
**Drinking status**					0.285
Never	1179	197 (16.7)	0.89 (0.65, 1.21)	0.456	
Ever	843	195 (23.1)	1.06 (0.79, 1.43)	0.693	

*Adjusted, if not stratified, for age, body mass index, total cholesterol, high density lipoprotein, triglycerides, smoking status and drinking status

## Discussion

Our study provides coherent evidence that there is a relationship between fasting glucose levels and the risk of the development of hypertension during a 15-year period among the Chinese rural women. We found that the baseline fasting glucose levels act as a risk predictor for the development of hypertension in non-hypertensive, Chinese rural women after adjusting for major risk factors. Moreover, our results demonstrated that TC significantly affected the association between fasting blood glucose and new onset hypertension. Hypercholesterolemia additively 176% increased the risk of new-onset hypertension for those with elevated fasting blood glucose levels. Even in normal TC levels female participants, 32% increased incidence of hypertension was occurred with fasting blood glucose levels elevation.

Several studies found that IFG significantly increased risk of new-onset hypertension [9–13], whereas a few studies showed no significant association [4, 14]. Shi et al. [9] firstly revealed the relationship between fasting blood glucose and risk of new-onset hypertension by a retrospective study conducted in a population with poor economic conditions. The Multi-Ethnic Study of Atherosclerosis (MESA) with 4.7 years of follow-up, demonstrated that the risk of hypertension increased by multi-fold with increased fasting glucose levels [10]. A cross-sectional survey study, conducted to evaluate the association of blood pressure with fasting blood glucose levels in Northeast China, demonstrated that fasting glucose was positively associated with BP in men, but not in women [11]. Another Japanese scholar Joshipura et al. [12] conducted a 5-year cohort study to show that higher baseline blood glucose level was an independent risk factor for new onset hypertension regardless of women and men. However, some controversy regarding the relationship between fasting glucose levels and risk of new-onset hypertension still occurs based on different adjustments covariates, such as age, obesity, lifestyle factors, obesity measurements, hyperuricemia and lipid profile, et al. [10, 15–19]. Consistent with our findings, Yang et al. conducted a 6-year cohort study in rural Chinese female adults, that results significantly supported that high fasting blood glucose levels lead to elevated blood pressure, and emphasizes the importance of IFG as an independent risk factor for the development of hypertension [13]. Complementary, our study further provides evidence that high baseline fasting blood glucose level is an independent risk factor for incidence of hypertension in Chinese rural women.

While the exact mechanism is unclear, the two conditions of hypertension and diabetes may share common plausible pathological mechanisms. For instance, as fasting glucose levels increase, advanced glycation end products (AGEs), reactive oxygen species (ROS), protein kinase C, and other oxidative or inflammation products, like malondialdehyde (MDA) and intercellular adhesion molecule-1 (ICAM-1) are formed and activated [20–24]. These products may disrupt homeostasis of endothelial and smooth muscle cells through activation of renin–angiotensin–aldosterone system (RAAS), thereby accelerating the damage to the vascular wall and kidney that lead to hypertension [25]. Additionally, the effect of hyperlipidemia on endothelial injury may be closely related to oxidized LDC (ox-LDL), which induces vascular endothelial cell dysfunction through a variety of pathways [26].

Our findings showed that rural women with IFG were more likely to develop new-onset hypertension, and the mechanism for the risk of hypertension with IFG differs by gender in Chinese population is not that clear. Imbalance of the above mechanisms due to differences in nutrition and lifestyle (smoking and drinking status) may explain greater future risk of developing hypertension in rural women with IFG.

However, there are still several limitations in this study. First, this study utilized part of data from a post-hoc analysis of a rural area osteoporosis cohort study, only new-onset hypertension information collected at baseline and at the end of follow-up was available for this analysis. Another weakness of the study was the lack of time of onset of hypertension. Therefore, it is possible that recall bias existed and the blood pressure levels of some participants who maybe occurred hypertension during the follow-up visit resumed normal levels afterward (because of some secondary hypertension or lifestyle factors change), leading to an bias in the assessment of the risk of hypertension. Second, all study participants were from a rural area, Anhui, in the east of China, therefore the study results may not be generalizable to other populations in China. Third, hypertension onset time could not be collected due to potential recall bias; this information would have helped to better predict the relationship between fasting blood glucose and risk of new-onset hypertension. Fourth, we did not obtain information on patient medication usage for hypercholesterolemia and/or a time variable, which was a confounding factor, to better predict the association between fasting blood glucose and new-onset hypertension. Finally, although oral glucose tolerance testing (OGTT) is an ideal method to evaluate impaired glucose metabolism (IGF), we were not able to conduct it. The test procedure is too complicated and expensive to perform in the rural as compared with fasting blood glucose measurement. Moreover, ambulatory blood pressure monitoring is one of the best methods for blood pressure evaluation, our study could not exclude some white-coat hypertension as well as masked hypertension as we measured blood pressure only at the local register center.

In the clinical prevention of hypertension, blood pressure variation should be carefully watched especially in women with increased fasting blood glucose levels even within normotensive conditions. Additionally, high TC levels may accelerate this process, especially in women with IFG.

## Data Availability

The datasets used and/or analyzed during the current study are available from the corresponding author on reasonable request.

## Abbreviations

CVD: Cardiovascular disease
RHR: Resting heart rate
eGFR: Estimated glomerular filtration rate
ADA: American Diabetes Association
IFG: Impaired fasting glucose
IGR: Impaired glucose regulation
DM: Diabetes mellitus
BP: Blood pressure
BMI: Body mass index
WHtR: Waist-to-height ratio
WHR: Waist-to-hip ratio
TC: Total cholesterol
TG: Triglycerides
HDL-C: High-density lipoprotein cholesterol
GEE: Generalized Estimating Equation
ORs: Odds ratios
CIs: Confidence intervals
MESA: Multi-Ethnic Study of Atherosclerosis
AGEs: Advanced glycation end products
ROS: Reactive oxygen species
MDA: Malondialdehyde
ICAM-1: Intercellular adhesion molecule-1
RAAS: Renin–angiotensin–aldosterone system
oX-LDL: Oxidized LDC
OGTT: Oral glucose tolerance testing
